# Photon coupling-induced spectrum envelope modulation in the coupled resonators from Vernier effect to harmonic Vernier effect

**DOI:** 10.1515/nanoph-2021-0596

**Published:** 2022-01-26

**Authors:** Lei Chen, Junhua Huang, Gui-Shi Liu, Feifan Huang, Huajian Zheng, Yaofei Chen, Yunhan Luo, Zhe Chen

**Affiliations:** Guangdong Provincial Key Laboratory of Optical Fiber Sensing and Communications, Department of Optoelectronic Engineering, College of Science and Engineering, Jinan University, Guangzhou 510632, China; Key Laboratory of Optoelectronic Information and Sensing Technologies of Guangdong Higher Education Institutes, Jinan University, Guangzhou 510632, China

**Keywords:** envelope multiplexing modulation, photon coupling, reference-free envelope modulation, silver nanowire, Vernier effect and harmonic Vernier effect

## Abstract

The Vernier effect and harmonic Vernier effect have attracted ever-increasing interest due to their freely tailored spectrum envelope in tunable laser, modulator, and precision sensing. Most explorations have mainly focused on configuring two isolated optical resonators, namely the reference and tunable resonator. However, this configuration requires a stable reference resonator to guarantee robust readout, posing a significant challenge in applications. Here, we discover the coupled-resonators configuration enabling a reference-free envelope modulation to address this problem. Specifically, all parameters of one resonator theoretically span a hypersurface. When the resonator couples to another one, photon coupling merit an escaped solution from the hypersurface, resulting in an envelope modulation independent of reference. We have first experimentally verified this mechanism in a coupled air resonator and polydimethylsiloxane resonator by inserting a semi-transparent 2-mercaptobenzimidazole-modified silver nanowire network. In addition, this novel mechanism provides a new degree of freedom in the reciprocal space, suggesting alternative multiplexing to combine more envelope modulations simultaneously. This study facilitates the fundamental research in envelope multiplexing. More importantly, the combination of silver nanowire network and flexible microcavity experimentally progress the spectral envelope modulation in optoelectronic integration inside resonators.

## 1 Introduction

The Vernier effect is a ubiquitous physical phenomenon to applications ranging from accurate sensing [1], [2], [3] to modulators in integrated photonics [4, 5] and microwave technology [6, 7]. Like a caliper, this effect utilizes an overlap response of two interferometers with slightly different frequencies to modulate the spectrum, thereby achieving a freely tailored envelope [8]. Precision control has always appealed to the advanced technology in fabrication and sensing. Hence, recent years have witnessed extensive applications benefiting from the Vernier effect, such as the tunable laser [9], [10], [11], [12], [13], frequency comb [14, 15], and various precision sensing [16], [17], [18], [19]. The harmonic Vernier effect is a recently extended notion from the Vernier effect [20], which generally requires one interferometer’s optical path length (OPL) to be integer multiples of the other. Benefiting from the large optical path difference, the harmonic Vernier effect merits a further amplified sensitivity than the Vernier effect [21].

So far, there are two mainstream configurations, i.e., parallel and series, commonly used to introduce the Vernier effect and harmonic Vernier effect [22]. In general, the former provides an advantage in interference visibility due to their crosstalk-free light intensities [23, 24]; the latter contributes a more compact packaging, widely used in space-starved chip applications [25, 26]. In principle, the summation of two resonators with similar spectrum generates the envelope modulation. To guarantee a robust readout, a controllable envelope needs a stable reference resonator and a tunable resonator. However, a stable reference spectrum is difficult to maintain in real applications. To be specific, for the parallel configuration, one needs an extra device to control the reference resonator’s physical parameters like temperature or stillstand; for the series configuration, the current trend in sensor fabrication pursues a compact design. Therefore, the reference resonator and tunable resonator are commonly closed to each other. In this manner, such sensor design has to sacrifice the stability of the reference resonator to trade off the compactness, which is particularly harmful for long time use.

As a parallel investigation, the Vernier effect and harmonic Vernier effect in the coupled resonators are of particular interest because of the elusive assumption to explain the experiments. For example, the conventional wisdom considers that the Vernier effect appears if two involved resonators possess similar OPL [27, 28]. In this manner, therefore, the coupled resonators must assume that the short resonator is part of a non-exist long resonator and omits the contribution from the short resonator [29, 30]. More confusingly, the harmonic Vernier effect happens when the OPLs of two resonators are comparable [31], [32], [33], [34], [35]. Still, such an effect appears if their OPL maintains multiple integers according to traditional theory. Alternatively, to avoid the theoretical conflict, several groups mathematically treated two coupled resonators as three reflectors [32, 36]. However, such treatment must assume that the counter-propagating light does not contribute to the output, breaking the Lorentz reciprocity [37].

This current study has clarified the elusive envelope modulation mechanism in the coupled resonators, and we spectacularly discovered that the photon coupling features a reference-free spectral envelope modulation, addressing the difficulty of the susceptible reference resonator. We are theoretically aware that the photon coupling enables an escaped solution from a hypersurface spanned from all possible parameters of one resonator, which results in a reference-free spectral envelope modulation. Then, this reference-free envelope modulation was experimentally verified by a pair of coupled resonators composing a length-tunable air resonator and a polydimethylsiloxane (PDMS) film resonator whose surface was coated with a surface-modified silver nanowire (AgNW)-based transparent heater to induce temperature perturbation stably. Equally importantly, an unprecedented high resolution of optical mode components was achieved in the reciprocal space, allowing us to observe the light distribution inside the resonators. This study has experimentally manifested that the coupled resonators configuration facilitates the optoelectronic integration and provides an extra degree of freedom in the reciprocal space, offering alternative multiplexing to combine and identify more modulated signals.

## 2 Results and discussion

### 2.1 Spectral envelop modulation in the coupled resonators

**Figure 1:**
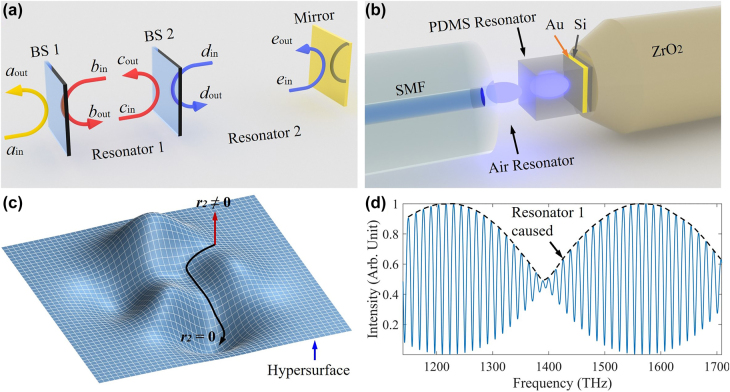
(a) Physical model of two coupled resonators. We mathematically treat two coupled resonators as two BSs and one mirror. (b) Structure of the coupled resonators composing a length-tunable air resonator and a length-fixed PDMS resonator. According to Fresnel’s formula, the calculated $r_{1}=\frac{n_{{\text{SiO}}_{2}}-n_{\text{Air}}}{n_{{\text{SiO}}_{2}}+n_{\text{Air}}}\approx 0.18$ and $r_{2}=\frac{n_{\text{PDMS}}-n_{\text{Air}}}{n_{\text{PDMS}}+n_{\text{Air}}}\approx 0.17$. (c) Hypersurface of Fabry–Pérot interferometer model, that is, non-modulated spectra. The black curve indicates that reflection varies with the parameters in resonator 2 when the length of resonator 1 is zero. The red curve indicates that the reflection escapes the hypersurface due to the photon coupling. (d) Envelope modulation depends on resonator 1 due to the photon coupling.

Here, we developed a new model based on the coupled-mode theory to exploit the spectrum modulation mechanism in the coupled resonators. Two coupled resonators were physically treated as two beam splitters (BSs) and one reflector, as shown in Figure 1a. In this theoretical framework, the light inside the resonators and input–output relationship can be written as [38],

(1) $$\left(\begin{array}{l} a_{\text{out}}\\ b_{\text{out}} \end{array}\right)=S_{1}\left(\begin{array}{l} a_{\text{in}}\\ b_{\text{in}} \end{array}\right)$$

(2) $$\left(\begin{array}{l} b_{\text{in}}\\ c_{\text{in}} \end{array}\right)=P_{1}\left(\begin{array}{l} c_{\text{out}}\\ b_{\text{out}} \end{array}\right)$$

(3) $$\left(\begin{array}{l} c_{\text{out}}\\ d_{\text{out}} \end{array}\right)=S_{2}\left(\begin{array}{l} c_{\text{in}}\\ d_{\text{in}} \end{array}\right)$$

(4) $$\left(\begin{array}{l} d_{\text{in}}\\ e_{\text{in}} \end{array}\right)=P_{2}\left(\begin{array}{l} e_{\text{out}}\\ d_{\text{out}} \end{array}\right)$$

(5) $$e_{\text{out}}=\eta e_{\text{in}}$$

where *a*_in_ and *a*_out_ are the input and output of resonator 1; *b* and *c* are the electric fields inside resonator 1, and *d* and *e* are the electric fields inside resonator 2; *η* is the effective reflectance, which considers the light beam diffusion loss depending on resonator length, and its initial value is *η*_Au_ ≈ 0.9 reflecting an experimental high-reflectance gold mirror; $S_{i}=\left(\begin{array}{ll} r_{i} & J_{i}\\ -J_{i} & r_{i} \end{array}\right)$ with the note of reflectance *r*_*i*_ and coupling *J*_*i*_; $P_{i}=\left(\begin{array}{ll} e^{j\varphi_{i}} & 0\\ 0 & e^{j\varphi_{i}} \end{array}\right)$, where *φ*_*i*_ = −4*πn*_*i*_*L*_*i*_/*λ*, medium refractive index *n*_*i*_, resonator length *L*_*i*_, and wavelength *λ*.

Then, the reflection can be expressed as,

$$R\equiv {\left|\frac{a_{\text{out}}}{a_{\text{in}}}\right|}^{2}$$

(6) $$={\left|r_{1}+\frac{J_{1}^{2}}{r_{1}-\frac{e^{j\varphi_{1}}}{r_{2}+\frac{J_{2}^{2}}{r_{2}-\frac{1}{\eta }e^{j\varphi_{2}}}}}\right|}^{2}.$$

A primary unit, $r_{i}+\frac{J_{i}^{2}}{r_{i}-\frac{1}{\eta }e^{j\varphi_{i}}}$, yields a periodic envelope modulation in the spectrum, and different from the conventional wisdom, both the photon coupling and reflectance contribute to the envelope modulation. Because of the photon coupling, the photons partly transmit from one resonator to the other. Consequently, the spectrum presents a primary unit nesting into the other, as shown in Eq. (6). Without loss of generality, we consider the most used high transmittance medium *r*_1_*r*_2_ << 1 and high-reflecting gold mirror *η*_Au_ ≈ 0.9. Then, Eq. (6) can be further simplified as (see Supplement Section II)

$$R\approx \eta^{2}+r_{1}^{2}+r_{2}^{2}-2r_{1}r_{2}\text{ cos}\left(\varphi_{1}\right)$$

(7) $$-2\eta r_{2}\text{ cos}\left(\varphi_{2}\right)+2\eta r_{1}\text{ cos}\left(\varphi_{1}+\varphi_{2}\right)$$

The reflection is composed of optical mode I cos(*φ*_1_), mode II cos(*φ*_2_), and mode III cos(*φ*_1_ + *φ*_2_), where modes II and III dominate the reflection in terms of *η* > *r*_1_, *r*_2_. Consequently, the slight phase difference *φ*_1_ between modes II and III results in the Vernier effect and harmonic Vernier effect. Although the intensity of mode I is relatively weak compared to modes II and III, mode I plays a vital role in the Vernier and harmonic Vernier effects. Because of mode I, the eigenvalues escape the hypersurface, yielding mode III together with mode II. Otherwise, the system eigenvalues are impossible to leave the hypersurface, thus without envelope modulation.

Notably, the reflection exists in the high dimensional parameter space spanned by all parameters include *η*, *r*_1_, *r*_2_, *φ*_1_, and *φ*_2_. A hypersurface with *r*_2_ = 0 is noteworthy because the reflection signifies a low Q-factor Fabry–Pérot interferometer no matter what value the other parameters take. Nevertheless, once the value of *r*_2_ ≠ 0, the reflection *R* escapes this hypersurface and obtains the envelope modulation, as shown in Figure 1c. More importantly, as shown in Figure 1d, such an envelope modulation obtained from the escaped hypersurface depends on *φ*_1_ merely, independent of any reference because modes II and III share a common phase component *φ*_2_.

### 2.2 Spectra evolution principle and confirmation experiments in the coupled resonators

We have implemented the confirmation experiments in the coupled resonators to demonstrate the new spectrum envelope modulation mechanism. The coupled resonators are composed of a length-tunable air resonator and a length-fixed PDMS film resonator. In experiments, we selected a silicon wafer size of 1 × 1 mm as the substrate to fabricate the resonators. A gold layer deposited the substrate with a thickness of ∼100 nm, forming a mirror with a ∼90% reflectance. Then, a layer of PDMS was coated on the gold mirror to form the film resonator. After that, we pasted the film resonator on a zirconium dioxide (ZrO_2_) bar via PDMS and mounted them on a stage with a step of 1.25 μm (Beijing Feichuang Yida Optoelectronics Technology, LPC20-60), corresponding to two positive pulses (see Figure S2a in the Supplement). When the film resonator was aligned to a coupler made by single-mode fiber, the gap between PDMS film and fiber forms the air resonator to trap photons, as shown in Figure 1b. We selected a supercontinuum source (SCS; OYSL, SC-5) with a spectrum ranging from 1.1 to 1.65 μm to probe the coupled resonators. An in-line polarizer connected the SCS to provide a linear polarization light input. The reflection from the coupled resonators was collected by a broadband 1 × 2 optical fiber splitter, and the spectrum was finally detected by an optical spectrometer analyzer (OSA; YOKOGAWA, AQ6370C) with a sampling rate of 0.1 nm.

**Figure 2:**
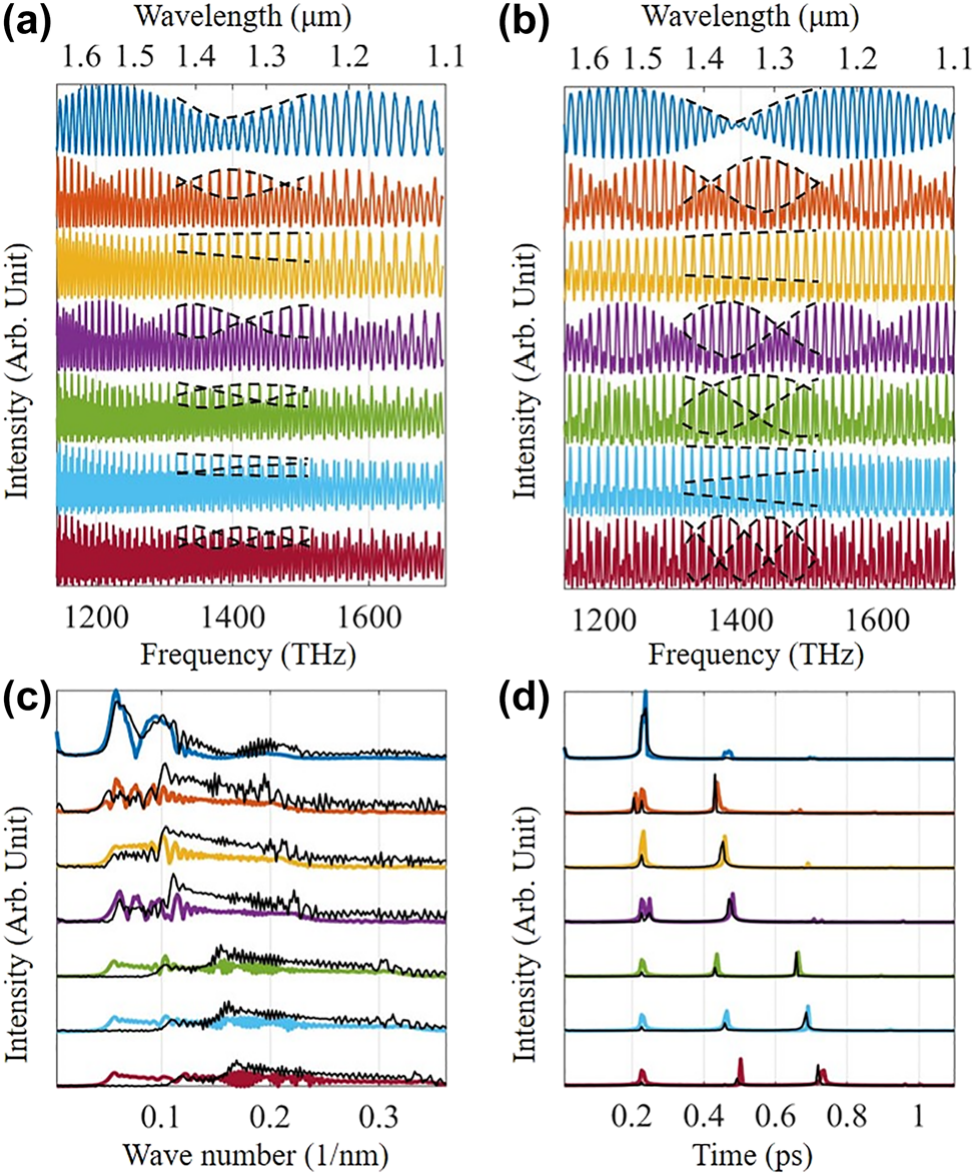
Typical reflections with envelope modulation in (a) experiments and (b) theory. From top to bottom, the reflections in (a) are the air resonator length 2.70, 65.20, 71.45, 77.70, 133.95, 141.45, and 151.45 μm. The reflections in (b) are the length of air resonator 2.70, 63.10, 69.58, 75.20, 131.00, 139.10, and 151.45 μm. The dashed curves guide the eye toward the modulation. Fourier transform shows the mode components in the reciprocal space of (c) wavelength and (d) frequency. The colored and black curves are the theoretical and experimental results.

The experiments and theoretical results are presented in Figure 2a and b. In experiments, the initial length of the air gap is ∼2.7 μm because of the manufacturing error of the 1 × 2 splitter (see Figure S3 in the Supplement). We carefully decreased the length of the air resonator to the minima. The experimental reflection spectrum in Figure 2a presents the Vernier effect, and the corresponding theoretical result in Figure 2b agrees with the experiments. Notice that the air resonator length is ∼2.7 μm, but the PDMS resonator length is ∼48.5 μm. Interestingly, the photon coupling generates the Vernier effect with highly different OPLs. In contrast, the conventional Vernier effect stems from cos[(*φ*_1_ + *φ*_2_)/2]cos[(*φ*_1_ − *φ*_2_)/2], requiring nearly equalized light path lengths.

The new mechanism likewise works on the harmonic Vernier effect. When the lengths of the two resonators are close to each other *φ*_1_ → *φ*_2_, the traditional wisdom expects the Vernier effect. However, because of the photon coupling, the OPL of mode III is twice that of mode II, yielding the first-order harmonic Vernier effect as the 2nd–4th curves shown in Figure 2a and b. Further increasing the OPL of the air resonator to twice the PDMS resonator, the second-order harmonic Vernier effect happens as the 5th–7th curves shown in Figure 2a and b.

Observation of the spectrum in the reciprocal space provides an intuitive framework to study the optical mode evolution. Figure 2c presents the mode evolution of the conventional Fourier transform (FT) in Figure 2a in the reciprocal space of wavelength. Unfortunately, the conventional approach merely gives the blur mode components [20, 39], and the experiments in Figure 2c verify this shortage. To improve the resolution, we revisited the fundamental notion of FT $f\left(t\right)=\frac{1}{\sqrt{2\pi }}\int F\left(\omega \right){\text{e}}^{i\omega t}\text{d}\omega$, where f(t) and F(ω) are the signal in the time domain and frequency domain, respectively. Note that the kernel ${\text{e}}^{i\omega t}$ is linear, but ω is inverse proportional to wavelength ω=2πc/λ. Therefore, it is impossible to obtain a nonlinear convolution kernel matching the signal with the uniform sampling at wavelength. Here, we implemented the FT after transforming the data with the uniform sampling at a wavelength to uniform sampling at angular momentum (see Supplement Section II). As a result, the matched sampling and convolution kernel dramatically increase the resolution in the reciprocal space, as shown in Figure 2d. Such an unprecedented high resolution allows us to gain insight into the mode evolution inside resonators.

**Figure 3:**
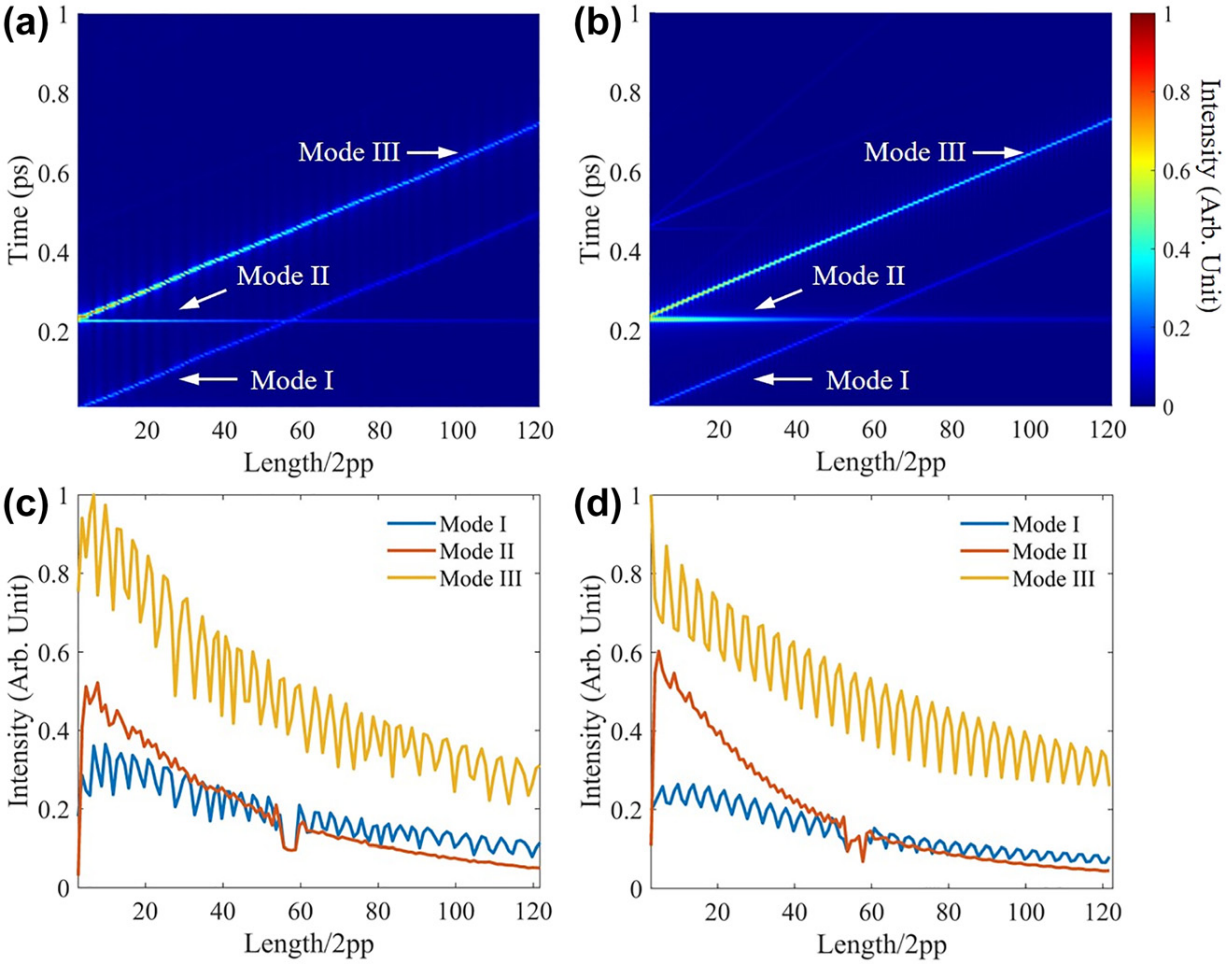
Mode evolution in the reciprocal space (a) experiments and (b) theory. The intensities of the three modes in (c) experiments and (d) theory. pp stands for the positive pulse, and 2pp equals 1.25 μm.

To comprehensively understand the envelope modulation mechanism in the coupled resonators, we have observed modes evolution via gradually increasing the air resonator length from 2.7 to 152.7 μm. Figure 3a and b provide the experimental and theoretical results in the reciprocal space, respectively. Three bright lines suggest the three involved optical modes in the envelope modulation. As expected in Eq. (7), mode II is the most apparent due to the PDMS resonator’s constant OPL. Mode I possesses a starting point closing origin, which refers to a near-zero air resonator length. The starting point at *φ*_1_ + *φ*_2_ indicates that the coupled resonator generates the optical mode III because of photon coupling. Likewise, due to photon coupling, modes I and III possess an identical slope as the length increase of the air resonator.

The intensity of modes I, II, and III depends on *r*_1_*r*_2_, *ηr*_2_, and *ηr*_1_, respectively. In terms of *η* > *r*_1_, *r*_2_, the intensity of mode I is minima, and that of mode III is maximal among the three modes initially. *r*_1_ is independent of the air resonator length, and *r*_2_ presents a weak dependence on the air resonator length (see Supplement Section II), resulting in a nearly unchanged *r*_1_*r*_2_. In contrast, the effective reflectance *η* considers light beam diffusion, and thus its value depends on the air resonator length (see Supplement Section II),

(8) $$\eta \left(z\right)=\eta_{\text{Au}}\frac{w_{0}}{w_{z}}\text{exp}\left(-\frac{r^{2}}{w_{z}^{2}}+\frac{r^{2}}{w_{0}^{2}}\right)$$

where *η*_Au_ = 0.9 and $w_{z}=w_{0}\sqrt{1+{\left[\lambda z/\left(\pi w_{0}^{2}\right)\right]}^{2}}$ is the waist evolution with the note of OPL *z* and beam radius *r*. Therefore, although three modes intensities exhibit the oscillation evolution, the expected values of modes II and III decay with the air resonator length, and mode I presents a weak dependence on the air resonator length compared to modes II and III, as shown in Figure 3c. However, due to the nearly unchanged *r*_1_*r*_2_ and rapidly decreased *ηr*_2_, the intensity of mode II finally overwhelms that of mode I, and the theoretical result confirm this signature in Figure 3d. In addition, we equally distributed modes I and II light intensity when their peaks cannot be distinguished around the crossing length ∼53 × 2pp, inducing sharply changed intensities. The theoretical results in Figure 3d presented a similar length happening the sharp change, verifying the experiments.

### 2.3 Reference-free spectral envelope modulation mechanism

**Figure 4:**
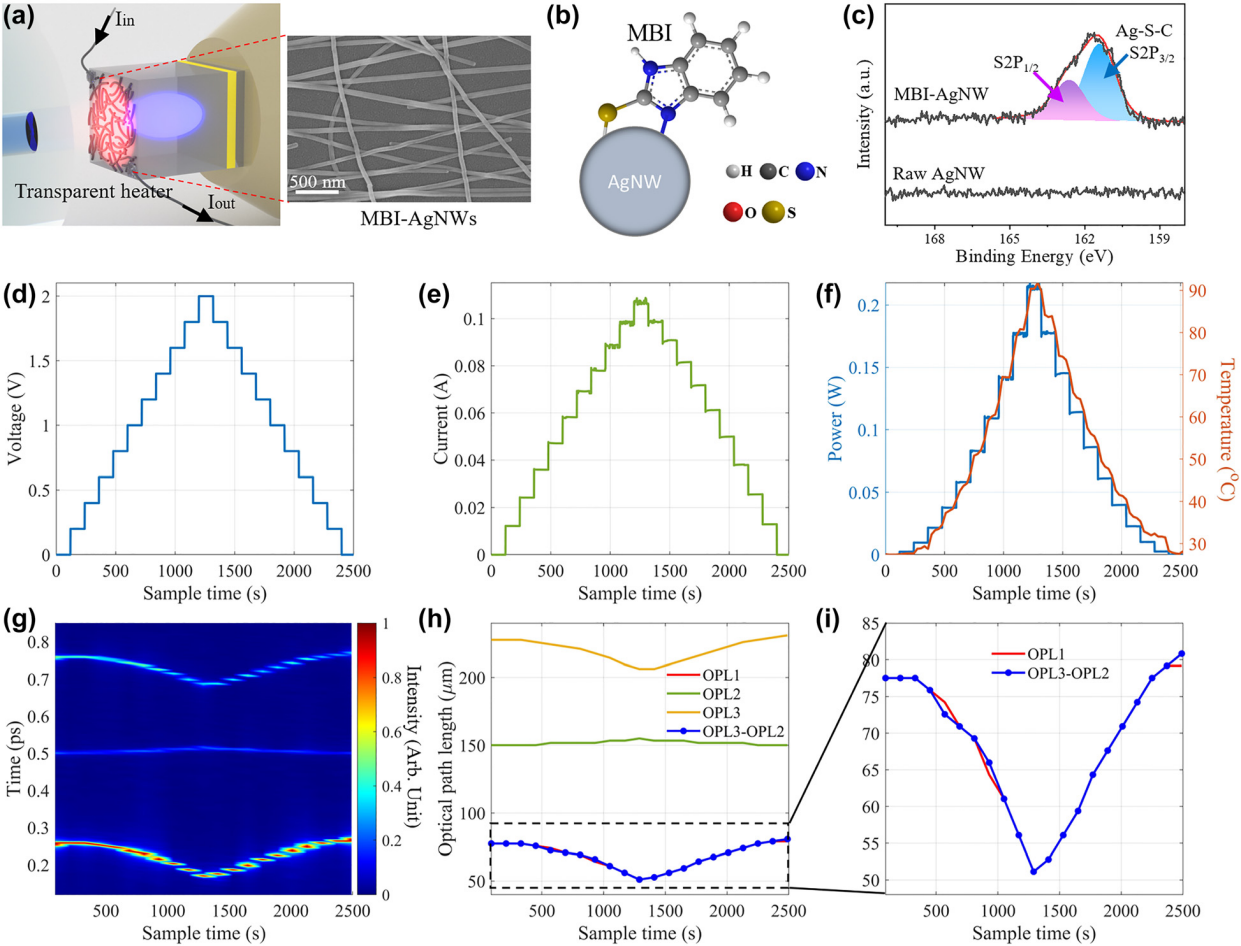
(a) Schematic illustration of the coupled resonators containing a AgNW-based transparent heater (left) and the scanning electron microscope (SEM) image of the MBI SAM-modified AgNW network on the PDMS film resonator (right). The cross-linked AgNW indicates a high-quality network. (b) Schematic of the AgNW modified with the MBI SAM via Ag–S and Ag–N bonds. (c) XPS S2P spectra of the raw AgNWs and MBI-modified AgNWs. A source meter applied voltage (d) and responded current (e) to the AgNW-based transparent heater. (f) The calculated power (left) and the corresponding temperature (right). (g) The mode components are observed from reciprocal space depending on the temperature. (h) The calculated OPL of modes I, II, and III. (i) Zoom in the OPL to compare the OPL1 and OPL3-OPL2.

We have implemented the confirmation experiments to verify the reference-free spectral envelope modulation mechanism in the coupled resonators. In the confirmation experiments, a AgNW-based transparent heater was fabricated on the PDMS film with a thickness of ∼106 μm, as shown in Figure 4a and S1 (see Supplement Section I). The AgNW-based heater can introduce the temperature perturbation into the coupled resonators and not obstruct the light transmission between the air resonator and the PDMS resonator. To improve the thermal stability of the heater, the AgNWs were modified by 2-mercaptobenzimidazole (MBI) via the Ag–S and Ag–N bonds to form a self-assembled monolayer (SAM) on the nanowire surface. Since the current density on individual nanowire is several orders of magnitude higher than that on the AgNW-based heater, and the non-uniform network inevitably induces local hot pots [40], the raw AgNW network would break down in a short time due to the electromigration and Plateau–Rayleigh instability. The MBI modification can effectively suppress the two side-effects on the AgNW [41], which improves the robustness of the transparent heater as the temperature perturbation source (see Figures S2 and S4). This modification was verified by the presence of S2P in the spectrum obtained from an X-ray photoelectron spectrometer (XPS) for the MBI-modified AgNWs (MBI-AgNW). After the modification, the temperature perturbation was stably yielded by applying step voltage. The applied voltage increased to 2 V from 0 V and gradually returned to 0 V with a step amplitude of 0.2 V, as shown in Figure 4b. The responded current and power presented a step fashion corresponding to the applied voltage, which results in the stepped temperature change with a maximum value of ∼90 °C, as shown in Figure 4c and d. The stepped temperature profile indicates the MBI-modified AgNW heater works well under the evaluated temperature (up to 90 °C) and enormous electrical stress with a maximum current density of 1200 mA/cm^2^.

Accordingly, the optical modes presented a similar stepped response, as shown in Figure 4g. Because the silicon wafer was pasted on the ZrO_2_ via a PDMS layer, the increased temperature expands the outside PDMS layer, which equally decreases the length of the air resonator. Consequently, Figure 4g shows modes I and III’s time dimension in the reciprocal space decrease as temperature increases. For mode II, the trend is reversed because the increased temperature enhances the PDMS resonator length. The calculated OPL of the three modes has verified this result, as shown in Figure 4h.

The OPL of mode III changing with that of mode II can verify the reference-free envelope modulation. As shown in Eq. (7), the envelope modulation in the coupled resonators is attributed to the OPL of modes II and III, and their difference equals the OPL of mode I due to the common phase component *φ*_2_. In this manner, if the OPL of mode II changes, it leads to an identical response to the OPL of mode III, forming the “following effect.” To confirm this effect, we calculated the optical path difference of modes III and II in Figure 4h. Figure 4i zoomed in on the interesting part of Figure 4h. Two nearly overlapped curves in Figure 4i present a similar response depending on the temperature, confirming the reference-free spectral envelope modulation mechanism. Meanwhile, note that Eq. (7) obtains modes I, II, and III under the condition of *r*_1_*r*_2_ << 1 and *η*_Au_ ≈ 0.9, and therefore, it is necessary to verify that this approximation is adequate in different OPL conditions. These overlapped curves have experimentally verified that the approximated condition is adequate when the OPL of the three modes change.

In addition, as a flexible material, the refractive index of the PDMS is temperature-dependent. Due to the extended temperature ranging from room temperature ∼25–∼90 °C, the changed PDMS refractive index alters the reflectance of the PDMS/Air interface. We calculated the refractive index of the PDMS under different temperatures according to an empirical formula [42]. As a result, the reflectance drops from ∼0.17 at 25 °C to ∼0.16 at 90 °C based on Fresnel’s formula. The reflectance only changes ∼−0.01 when the temperature dramatically increases from 25 to 90 °C. Here, we neglected the limited influence of the temperature on the reflectance, and the experiments under different temperatures also confirmed the nearly unchanged reflectance.

### 2.4 Envelope multiplexing modulations

Multiplexing is a desirable technology for parallel sensing simultaneously. Nevertheless, the sensing application based on the Vernier effect or harmonic Vernier effect works when one set of spectral envelope modulation is leveraged [43, 44]. The crosstalk from another envelope modulation may ruin both envelopes because the conventional method lacks a dimension to demodulate the signals. For example, two sets of envelope modulations in the conventional Vernier effect are composed of cos[(*φ*_1_ + *φ*_2_)/2], cos[(*φ*_1_ − *φ*_2_)/2], cos[(*φ*_3_ + *φ*_4_)/2], and cos[(*φ*_3_ − *φ*_4_)/2], where cos[(*φ*_1_ − *φ*_2_)/2] and cos[(*φ*_3_ − *φ*_4_)/2] stands for the envelope. In the case *φ*_1_ ≈ *φ*_2_ and *φ*_3_ ≈ *φ*_4_, the Vernier effect is most distinguishable for both groups. Unfortunately, we cannot identify their mode components because of the nearly overlapped cos[(*φ*_1_ − *φ*_2_)/2] and cos[(*φ*_*3*_ − *φ*_*4*_)/2]. In contrast, the mode II of coupled resonators serves as the baseline of mode III, potentially offering a new degree of freedom in the reciprocal space to separate different envelope modulations.

**Figure 5:**
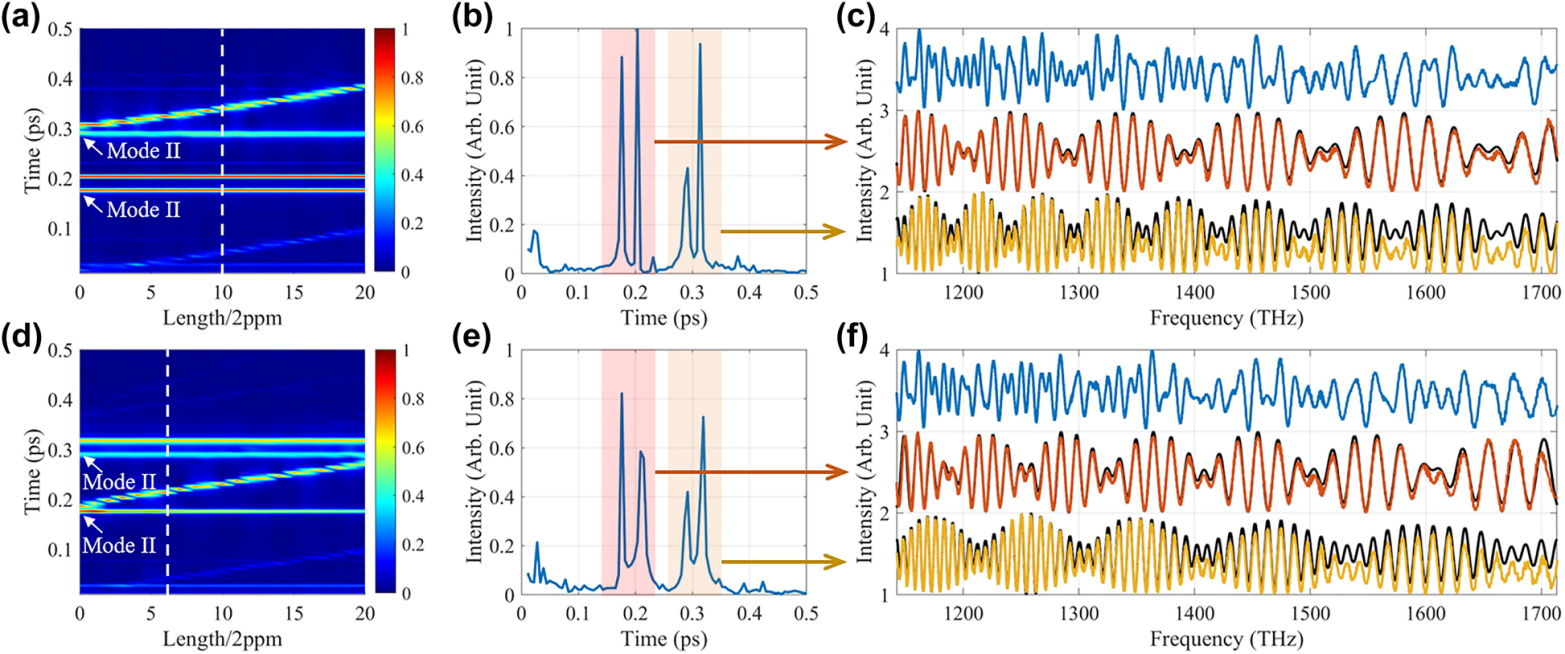
Experimental envelope multiplexing modulation of two sets of coupled resonators with (a) a fixed air resonator length of ∼13.2 μm in the thin PDMS resonator group, and we changed air resonator length in the thick PDMS resonator group. (b) A typical spectral component in the reciprocal space corresponds to the dashed white line in (a), where the air resonator length changes 12.5 μm. (c) The blue curve corresponds to experimental reflections from two coupled resonators. The curves with red and orange are the measured reflections individually, and the black curves are the demodulated signals via extracting the mode components inside the window of (b). (d)–(f) maintain a similar meaning to (a)–(c). In (d), the fixed air resonator length was ∼8.3 μm in the thick PDMS resonator group, and we changed air resonator length in the thin PDMS resonator group. (e) A typical spectral component in the reciprocal space corresponds to the dashed white line in (d), where the air resonator length changes 7.5 μm.

We have conducted proof-of-concept experiments with two sets of coupled resonators to verify this advantage (set up in the Supplement). Two PDMS resonators have a thickness of ∼38 and ∼62 μm, corresponding to 0.18 and 0.29 ps in Figure 5a. We maintained the air resonator length of ∼13.2 μm in the thin PDMS resonator group and changed the air resonator length in the thick PDMS resonator group. The evolutive modes are presented in Figure 5a. The unchanged mode III > 0.2 ps indicates the constant air resonator length of the thick PDMS resonator group, and the tilted mode III suggests the varied air resonator length of the thin PDMS resonator group.

The baselines of these two groups maintain during the test to identify multiplexing. We take an example of a typical air resonator length of ∼15.2 μm in the thick PDMS resonator group to show the demodulation, where the write dashed line labels in Figure 5a. FT result of air resonator length of ∼15.2 μm is plotted in Figure 5b. The blue curve in Figure 5c shows the measured spectrum from two coupled resonators, and the spectrum indeed presents a damaged envelope, which cannot use in sensing. Then, we carried out the inverse FT after applying a bandpass filter to select one set of modes II and III, as shown in Figure 5b, and the black curves in Figure 5c demonstrate the inverse FT results. The red and orange curves were the measured spectra when we removed one of the two sets of coupled resonators. The measured reflection nearly overlaps with the recovery reflections, verifying the multiplexing and demultiplexing in the coupled resonator configuration. After that, similar results are obtained in Figure 5d–f if the air resonator length of the thick PDMF resonator group maintains and changes the air resonator length of the thin PDMF resonator group.

## 3 Conclusions

This current work presents that the coupled resonator endows a new mechanism of the Vernier effect and harmonic Vernier effect. We discovered that the photon coupling generates an escaped reflection from the hypersurface spanned the parameters of one resonator. As a result, this new mechanism merits the spectral envelope modulation independently of the reference resonator. The confirmation experiment using the MBI-modified AgNW transparent heater as an electric controller has manifested this promising approach in optoelectronic integration. In addition, this new mechanism provides a robust carrier in the reciprocal space, which offers a new multiplexing approach to integrate more envelope modulations.

Equally importantly, we have shown a new technique, generating an unprecedented high resolution in the reciprocal space for observing the optical modes evolution. The experiments have first manifested the optical mode evolution from the Vernier effect to the harmonic Vernier effect in the reciprocal space. The arrangement of coupled resonators provides an encouraging method for the Vernier effect and harmonic Vernier effect.

## Supplementary Material

Supplementary Material
