# Supplementary material for: Photon coupling-induced spectrum envelope modulation in the coupled resonators from Vernier effect to harmonic Vernier effect
Source: Nanophotonics. 2022 Jan 26;11(5):957–66. doi: 10.1515/nanoph-2021-0596 (PMC11501918; doi:10.1515/nanoph-2021-0596)
Supplement: Supplementary file 1 — Supplementary Material [file j_nanoph-2021-0596_suppl.docx]

Lei Chen, Junhua Huang, Gui-Shi Liu^*^, Feifan Huang, Huajian Zheng, Yaofei Chen, Yunhan Luo^*^, and Zhe Chen^[[1]](#footnote-1)^

**Photon coupling-induced spectrum envelope modulation in the coupled resonators from Vernier effect to harmonic Vernier effect**

1. Resonator preparation, characterization, and experimental setup


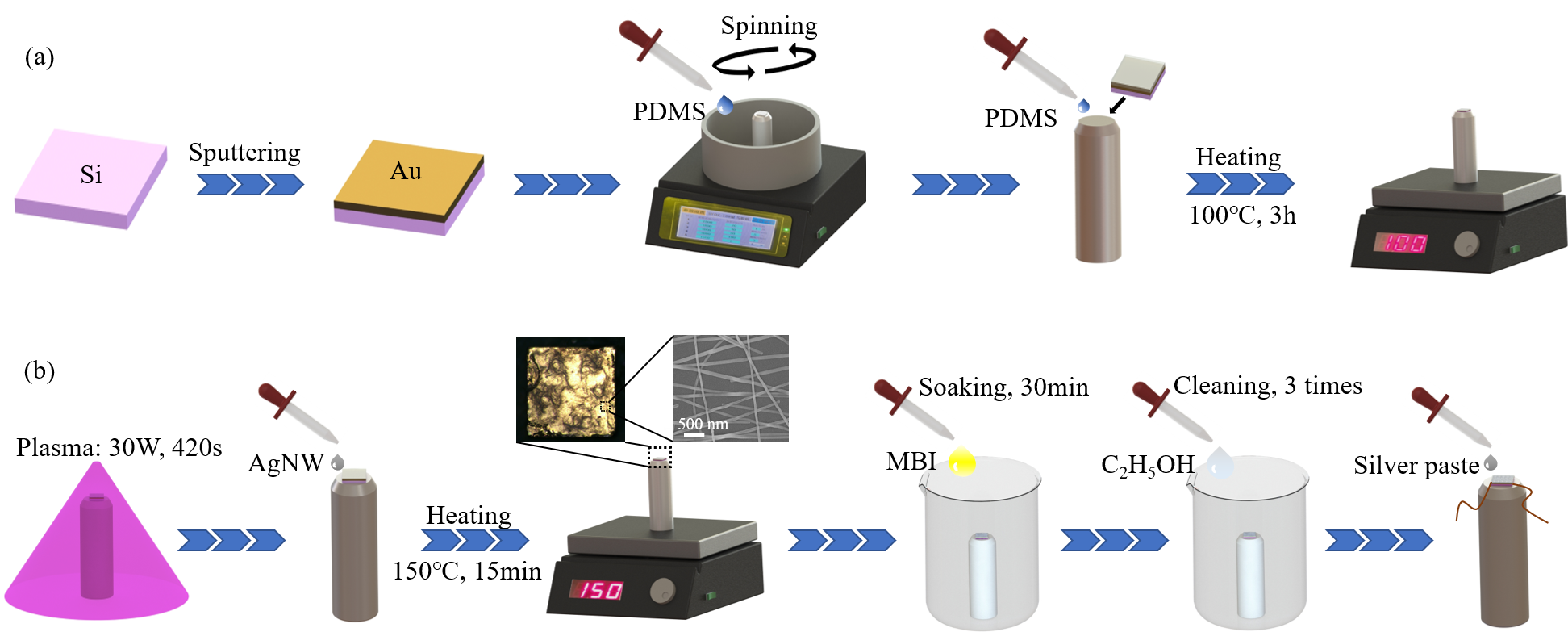


Fig. S1. (a) The fabrication process of the PDMS resonator. (b) The fabrication process of the MBI-AgNW-based heater.

**PDMS resonator fabrication.** A silicon wafer (1 × 1 mm^2^) was used as the substrate for fabricating the PDMS resonator. A chromium layer with 5 nm thickness and a gold layer with 100 nm thickness were successively deposited on the silicon substrate by a vacuum deposition machine (ZZS-700B, Chengdu Vacuum Machinery Inc.), where the chromium layer was to improve the adhesion strength of the gold layer to the silicon substrate (Au/Si). The PDMS base and curer (w/w = 10:1) mixture were degassed in a vacuum chamber for 1 h and spin-coated on the Au/Si substrate. After that, we pasted the film resonator on a zirconium dioxide (ZrO_2_) bar via PDMS. Then, the PDMS was thermally cured at 100 °C for 3 h.

**AgNW heater fabrication.** The PDMS surface was firstly pretreated by air plasma to improve surface hydrophilicity (SUNJUNE PLASMA VP-R5). Then, Ethanol-dispersed AgNWs with an average diameter of 90 nm and length of ~20 µm (purchased from Zhejiang Kechuang Inc) were prepared. The dispersion was agitated and diluted by ethanol to a concentration of w_Ag_/w_ethanol_ = 1:2. The diluted solution was coated to form an AgNW network on the PDMS film under the condition of 150 ℃ within 15 min. After drying, the AgNW film was immersed into the MBI solution for surface modification, where MBI powder was dissolved in ethanol at a concentration of 0.1 M. After the modification and rinsing with ethanol, two silver threads were attached to the opposite corners of the MBI-modified AgNW network using the silver paste to construct the heaters.

**Characterization.** An SEM (Carl Zeiss SUPRA 60) and OM were used for the morphology characterization of AgNWs. An X-ray photoelectron spectrometer (Thermo Scientific K-Alpha X) was used to verify the modification of AgNWs with MBI. The AgNW heater was powered by a source meter (Keithley 2611b). The resultant temperature was monitored by an infrared camera (FOTRIC, 228s).

**Experimental setup.** As shown in Fig. S2(a), we mounted the ZrO_2_ bar on a stage with a step of 1.25 μm (Beijing Feichuang Yida Optoelectronics Technology, LPC20-60), corresponding to two positive pulses (pp). When the film resonator was aligned to a coupler made by single-mode fiber, the gap between film and fiber forms the air resonator to trap photons. The optical microscope picture of the broadband 1×2 fiber splitter, as shown in Fig. S3. An uneven surface can be found at the then of the splitter, and it causes ~2.7 μm initial length of the air resonator. Then, we employed a supercontinuum source (SCS; OYSL, SC-5) with a spectrum ranging from 1.1 to 1.65 μm to probe the coupled resonators. An in-line polarizer connected the SCS to provide a linear polarization light input. The reflection from the coupled resonators was collected by the splitter, and it was finally detected by an optical spectrometer analyzer (OSA; YOKOGAWA, AQ6370C) with a sampling rate of 0.1 nm. In Fig. S2(b), a source meter was used to change the temperature inside the resonator. Then, in Fig. S2(c), another resonator was added to test the multiplex and demultiplex of the coupled resonators.


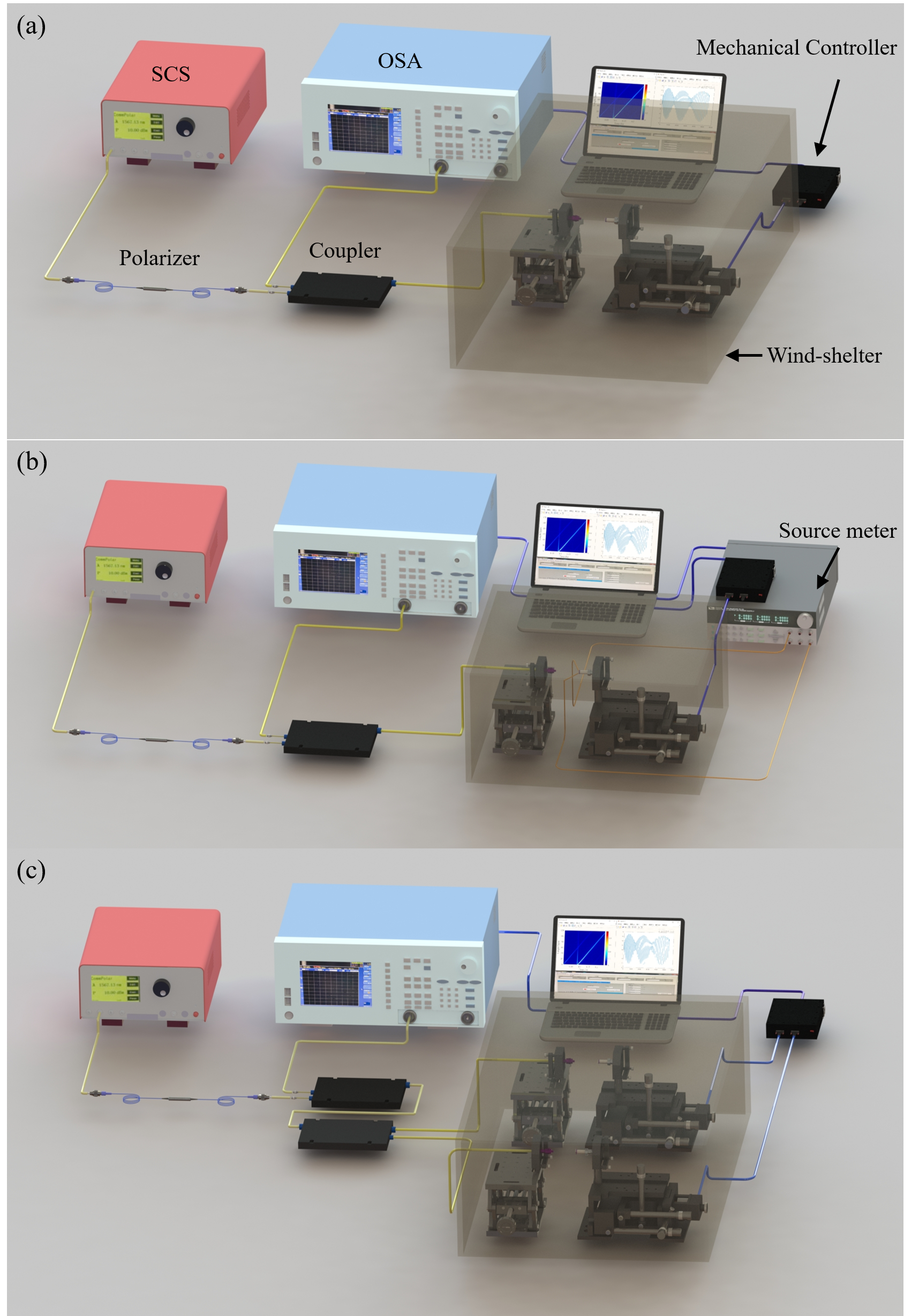


Fig. S2. Experimental setup (a) in Figs. 2 and 3, (b) in Fig. 4, and (c) in Fig. 5.


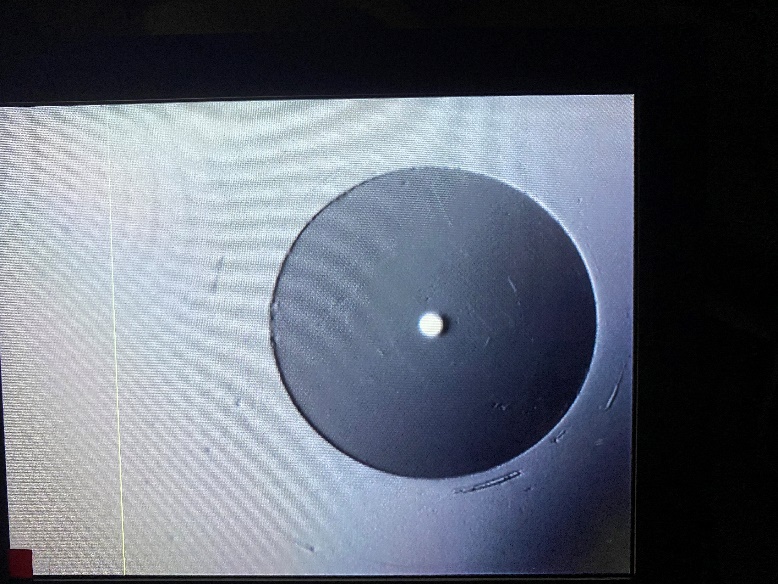


Fig. S3. Optical microscope picture of the broadband 1×2 fiber splitter.

1. Derivations and Calculation of reciprocal space in the frequency
2. Derivations of $\eta\left( z \right)$ and $r_{2}\left( z \right)$

Suppose the light inside FP resonator obeys the Gaussian distribution. Then, it can be written as,

$I\left( r \right)=\frac{2P}{\pi w_{z}^{2}}\exp\left( -\frac{2r^{2}}{w_{z}^{2}} \right)$

where $w_{z}=w_{0}\sqrt{\left( 1+\left( \frac{\lambda z}{\pi w_{0}^{2}} \right)^{2} \right)}$ and $z=L_{1}+L_{2}$. The initial light intensity is described as,

$I_{0}\left( r \right)=\frac{2P}{\pi w_{0}^{2}}\exp\left( -\frac{2r^{2}}{w_{0}^{2}} \right)$.

We defined the $\eta\left( z \right)$ as

$\eta\left( z \right)=\eta_{Au}\sqrt{\frac{I}{I_{0}}}=\eta_{Au}\frac{w_{0}}{w_{z}}\exp\left( -\frac{r^{2}}{w_{z}^{2}}+\frac{r^{2}}{w_{0}^{2}} \right)$

where $\eta_{Au}=0.9$.

With the same reason, $r_{2}$ is dependent on the air cavity length $L_{1}$. Therefore, $r_{2}\left( L_{1} \right)$ can be written as,

$$r_{2}\left( z \right)=\frac{w_{0}}{w_{L_{1}}}\exp\left( -\frac{r^{2}}{w_{L_{1}}^{2}}+\frac{r^{2}}{w_{0}^{2}} \right)$$

where $w_{L_{1}}=w_{0}\sqrt{\left( 1+\left( \frac{\lambda L_{1}}{\pi w_{0}^{2}} \right)^{2} \right)}$

1. Derivations of *R*

$$R=\left| \frac{\eta-\eta r_{1}r_{2}e^{i\varphi_{1}}-r_{2}e^{i\varphi_{2}}+r_{1}e^{i\left( \varphi_{1}+\varphi_{2} \right)}}{\eta r_{1}-\eta r_{2}e^{i\varphi_{1}}-r_{1}r_{2}e^{i\varphi_{2}}+e^{i\left( \varphi_{1}+\varphi_{2} \right)}} \right|^{2}$$

Considering *η≤0.9,* *r_1_*≈0.18, *r_2_*≈0.17, the terms of $\eta r_{1}-\eta r_{2}e^{i\varphi_{1}}-r_{1}r_{2}e^{i\varphi_{2}}\ll e^{i\left( \varphi_{1}+\varphi_{2} \right)}$ and $\eta r_{1}r_{2}e^{i\varphi_{1}}\ll\eta-r_{2}e^{i\varphi_{2}}+r_{1}e^{i\left( \varphi_{1}+\varphi_{2} \right)}$. Thus,

$$R\approx\left| \left( \eta e^{-i\varphi_{2}}-r_{2} \right)e^{-i\varphi_{1}}+r_{1} \right|^{2}$$

$$R=\eta^{2}+r_{1}^{2}+r_{2}^{2}-2r_{1}r_{2}\cos\left( \varphi_{1} \right)-2\eta r_{2}\cos\left( \varphi_{2} \right)+2\eta r_{1}\cos\left( \varphi_{1}+\varphi_{2} \right)$$

1. Calculation of reciprocal space in the frequency


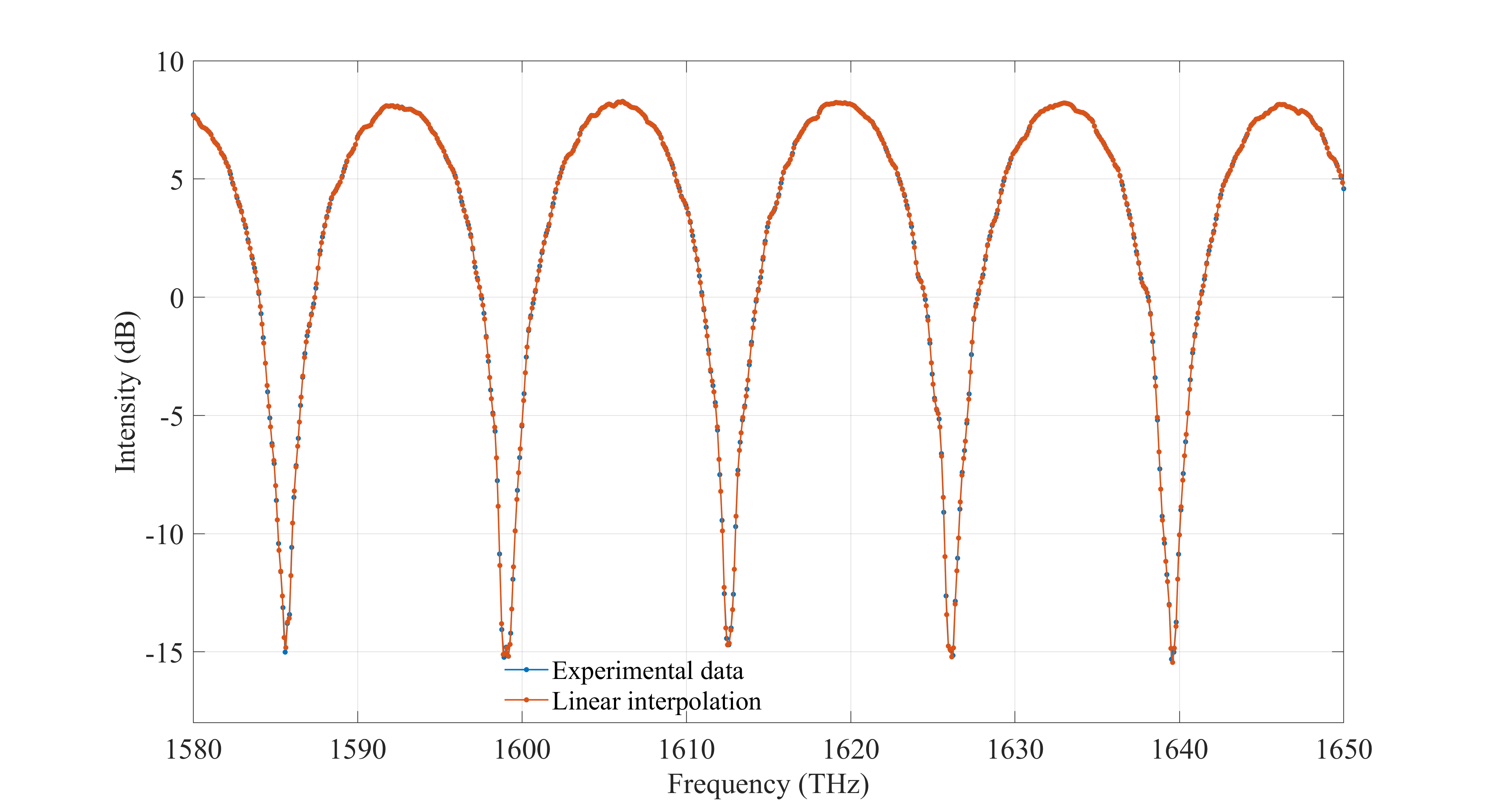


Fig. S4. Demonstration of the experimental data and after linear interpolation.

The conventional Fourier transformation to a spectrum is applied to the wavelength, $Y\left( t \right)=\frac{1}{2\pi}\int y\left( \lambda\right)e^{-i2\pi\frac{c}{\lambda}t}d\lambda$. Therefore, the dispersion is inevitable because the wavelength place at the denominator. In this study, we employed the frequency as the kernel, $Y\left( t \right)=\frac{1}{2\pi}\int y\left( f \right)e^{-i2\pi ft}df$. Consequently, the impact of the dispersion is removed. However, before this mathematical treatment, a linear interpolation must be applied to satisfy the kernel, and the result can be found in Fig. S3. The experimental data and linear interpolation are overlapped, indicating the undistorted signal processing.

1. Performance comparison of AgNW heater with and without MBI modification


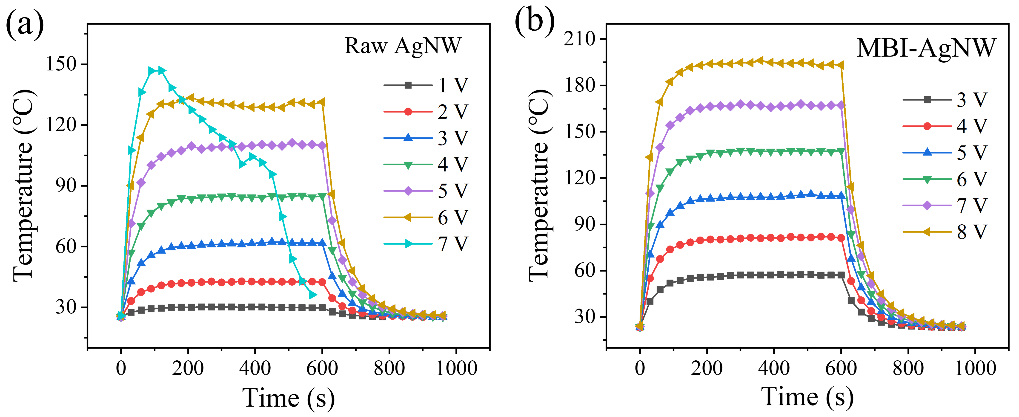


Fig. S4. Temperature profiles of (a) raw AgNW and (b) MBI-modified AgNW (MBI-AgNW) heaters on a glass of 20 × 30 mm^2^ under different applied voltages.

**Raw AgNW V.S. MBI modified AgNW.** The surface temperatures of the two heaters reach saturation values when the Joule heating balances with the power loss. Therefore, a larger voltage leads to a higher saturation temperature. When applied with 7 V, the raw AgNW heater yields a maximum temperature of 150 °C within 100 s but rapidly exhibits a decrease of temperature, indicating the percolative paths of the AgNW network are partly destroyed. By contrast, the MBI-modified AgNW heater can withstand a higher temperature under 8 V without any degradation. The improved stability arises from the MBI modification, which can introduce interface viscous force to suppress surface atom diffuse of the modified AgNWs under the Joule heating and electromigration.

1. ***Corresponding author: Gui-Shi Liu, Yunhan Luo,** Guangdong Provincial Key Laboratory of Optical Fiber Sensing and Communications, Department of Optoelectronic Engineering, College of Science and Engineering, Jinan University, Guangzhou 510632, China, e-mail: [guishiliu@163.com](mailto:guishiliu@163.com) (G. Liu), yunhanluo@163.com (Y. Luo)

   **Lei Chen, Junhua Huang, Feifan Huang, Huajian Zheng, Yaofei Chen, and Zhe Chen,** Guangdong Provincial Key Laboratory of Optical Fiber Sensing and Communications, Department of Optoelectronic Engineering, College of Science and Engineering, Jinan University, Guangzhou 510632, China**;** Key Laboratory of Optoelectronic Information and Sensing Technologies of Guangdong Higher Education Institutes, Jinan University, Guangzhou 510632, China [↑](#footnote-ref-1)
